# Aging characteristics of asphalt binders modified with waste tire and plastic pyrolytic chars

**DOI:** 10.1371/journal.pone.0256030

**Published:** 2021-08-19

**Authors:** Abhinay Kumar, Rajan Choudhary, Ankush Kumar

**Affiliations:** Department of Civil Engineering, Indian Institute of Technology Guwahati, Guwahati, Assam, India; Beijing University of Technology, CHINA

## Abstract

Globally, the growing volume of waste tires and plastics has posed significant concerns about their sustainable and economical disposal. Pyrolysis provides a way for effective treatment and management of these wastes, enabling recovery of energy and produces solid pyrolytic char as a by-product. The use of pyrolytic chars in asphalt binder modification has recently gained significant interest among researchers. As asphalt binder aging influences the cracking, rutting, and moisture damage performance of asphalt binder and the mixtures, evaluation of aging characteristics of char modified asphalt binders is quite important. The main objective of this study is the investigation of the aging characteristics of asphalt binders modified with waste tire pyrolytic char (TPC) and waste plastic pyrolytic char (PPC) through rheological and spectroscopic evaluations. To imitate short-term and long-term aging conditions, the asphalt binders were first treated in a rolling thin film oven (RTFO) and then in a pressure aging vessel (PAV). The aging characteristics were determined using four rheological aging indices based on complex modulus (G*), phase angle (δ), zero shear viscosity (ZSV), and non-recoverable creep compliance (*J*_*nr*_) from multiple stress creep and recovery (MSCR) test. The fatigue cracking potential was then measured through binder yield energy test (BYET). These parameters were measured through a dynamic shear rheometer. Fourier transform infrared (FTIR) and proton nuclear magnetic resonance (^1^H-NMR) spectroscopy analyses were then used to investigate changes in chemical composition due to aging in the char modified binders. Both TPC and PPC improved the high-temperature deformation resistance properties of asphalt binder. The TPC-modified binder showed better aging resistance than the control and PPC-modified binders, based on the different rheological and spectroscopic indices. The pyrolytic char modified binders also demonstrated good fatigue performance.

## Introduction

About 1.4 billion tires reach the end of life and become waste each year globally [1]. Consequently, the disposal of scrap tires is an increasing environmental and ecological problem. Plastic wastes are produced in large quantities due to their heavy demand in diverse sectors such as packaging, automobiles, electronics, household, agriculture, and other applications [2,3]. Therefore, the sustainable disposal of tire and plastic wastes has become a significant concern in many countries, including India. Concerted efforts are being made to develop sustainable and energy-efficient processes for treating and processing end-of-life tires and plastics. Pyrolysis technology has gained enormous research interest in recent years as an interesting thermochemical route to address the treatment of waste tires and plastics. The products of the pyrolysis process include liquid pyrolytic oil, gases, and solid char. Some factors influencing the composition of pyrolytic products are raw material, pyrolysis reaction conditions, reactor type, and catalyst [4].

Asphalt binder (also called bitumen) is the binding agent used for road pavements, parking areas, and driveways worldwide. Chemically, asphalt binder consists of a large number of various types of organic compounds, ranging from paraffins to alkyl polyaromatics containing heteroatoms (N, O, S) and metal traces (vanadium, nickel, iron). During its service life, asphalt binder is subjected to a series of complex physio-chemical processes such as oxidization, volatilization, condensation, polymerization, thixotropy (or steric hardening), causing the binder to become stiffer (harder) and brittle [5–8]. This phenomenon is termed asphalt aging. A highly aged asphalt binder (or asphalt mixture) may mobilize an early onset and propagation of pavement distresses such as fatigue cracking, thermal cracking, and moisture damage [8,9]. Asphalt binder ages in two phases: (1) short-term aging (manifests during production, placement, and compaction of the asphalt mixture), and (2) long-term aging (manifests during the service life of the asphalt pavement when exposed to the proximate environment).

Many wastes and by-product materials such as plastic wastes, crumb rubber, slags, and crushed concrete have previously been explored as alternative additives to asphalt binder or hot mix asphalt [10,11]. While the tire and plastic pyrolytic oils find applications in energy generation and gases are reused for their heat value, the solid carbonaceous char produced in pyrolysis is regarded as a by-product. There is a growing need to finds routes for its broader utilization in bulk quantities. Several carbonaceous materials have also been utilized in previous research studies, such as biochar, carbon black, and carbon fibers [12–14]. In recent years, some studies have reported the use of tire pyrolytic char (TPC) in asphalt binder modification and evaluated its effect on binder physical properties, binder rheology, and mixture properties [15–19]. The use of plastic pyrolytic char (PPC) for asphalt modification has also recently gained interest [20,21].

Considering the need and importance of evaluation of aging characteristics for a modified asphalt binder, only limited studies have been done on the characterization of aging behavior of an asphalt binder with TPC modification. No study was found on the evaluation of the aging properties of PPC modified binders. Feng et al. [16] evaluated the aging behavior of asphalt binders modified with tire pyrolytic carbon black (PCB) employing penetration, ductility, softening point, and viscosity tests (four aging indices were formulated) before and after short-term and long-term thermo-oxidative aging, and photo-oxidative aging. The results showed that PCB modified binders exhibited better aging resistance than unmodified binders based on all four aging indices. In another study, Wang et al. [18] assessed the aging properties of modified asphalt containing tire vacuum pyrolysis derived carbon black using multiple stress creep and recovery (MSCR) tests. A single aging index was formulated based on the ratio of non-recoverable creep compliance (*J*_*nr*_) before and after short-term and long-term aging. The MSCR *J*_*nr*_ results showed improvement in aging resistance with further improvements at higher PCB contents. As observed from the literature review, although some efforts have been made to characterize aging properties with TPC modified binders, works directed to comprehend the aging properties considering both rheological and spectroscopic techniques are still quite limited. TPC and PPC originate from quite different raw materials, hence it is expected that they will have differing effects on the physio-chemical and aging properties of the resulting modified binders. No comparative study is available to understand and compare the aging properties of asphalt binders modified with char from the pyrolysis of two abundant waste materials (waste tire and waste plastic).

This study aims to evaluate the effects of aging on the rheological and spectroscopic properties of asphalt binders modified with chars derived from the pyrolysis of waste tires and waste plastics. To detect changes in rheological parameters and chemical composition under various aging states, a dynamic shear rheometer (DSR), Fourier transform infrared (FTIR), and proton nuclear magnetic resonance (NMR) spectroscopies were employed in this study. Frequency sweep tests were performed to obtain linear viscoelastic properties (complex modulus and phase angle) and zero shear viscosity (ZSV). MSCR test was conducted to understand the high-temperature deformation resistance of binders in the nonlinear viscoelastic regime. Aging indices were then formulated based on rheological tests and FTIR analysis to quantitatively analyze the changes occurring in the modified and control binders due to aging. Long-term aged binders were also subjected to binder yield energy test (BYET) to assess the effect of aging on the fatigue resistance of the long-term aged binders.

**Fig 1:**
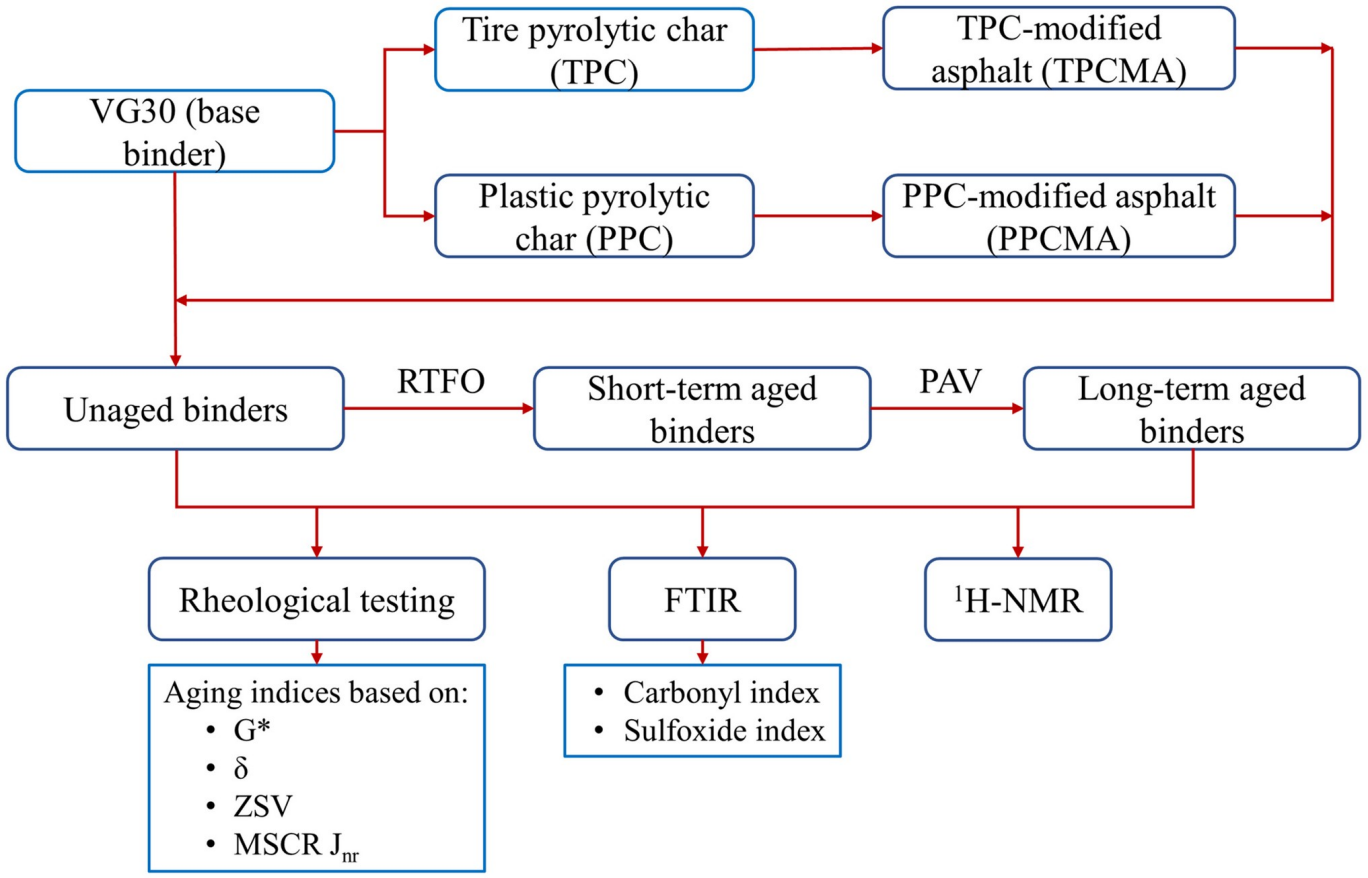
Experimental flowchart.

Fig 1 shows the flowchart with an overview of the materials and research plan used in this study. The details of the materials (base binder, TPC, and PPC), preparation of modified binders, description of rheological and spectroscopic tests, aging indices, and the corresponding results and discussion are presented in the subsequent sections of this article. The research article then concludes with a summary of important findings, contributions, and recommendations.

## Materials and methodology

### Materials

**Fig 2:**
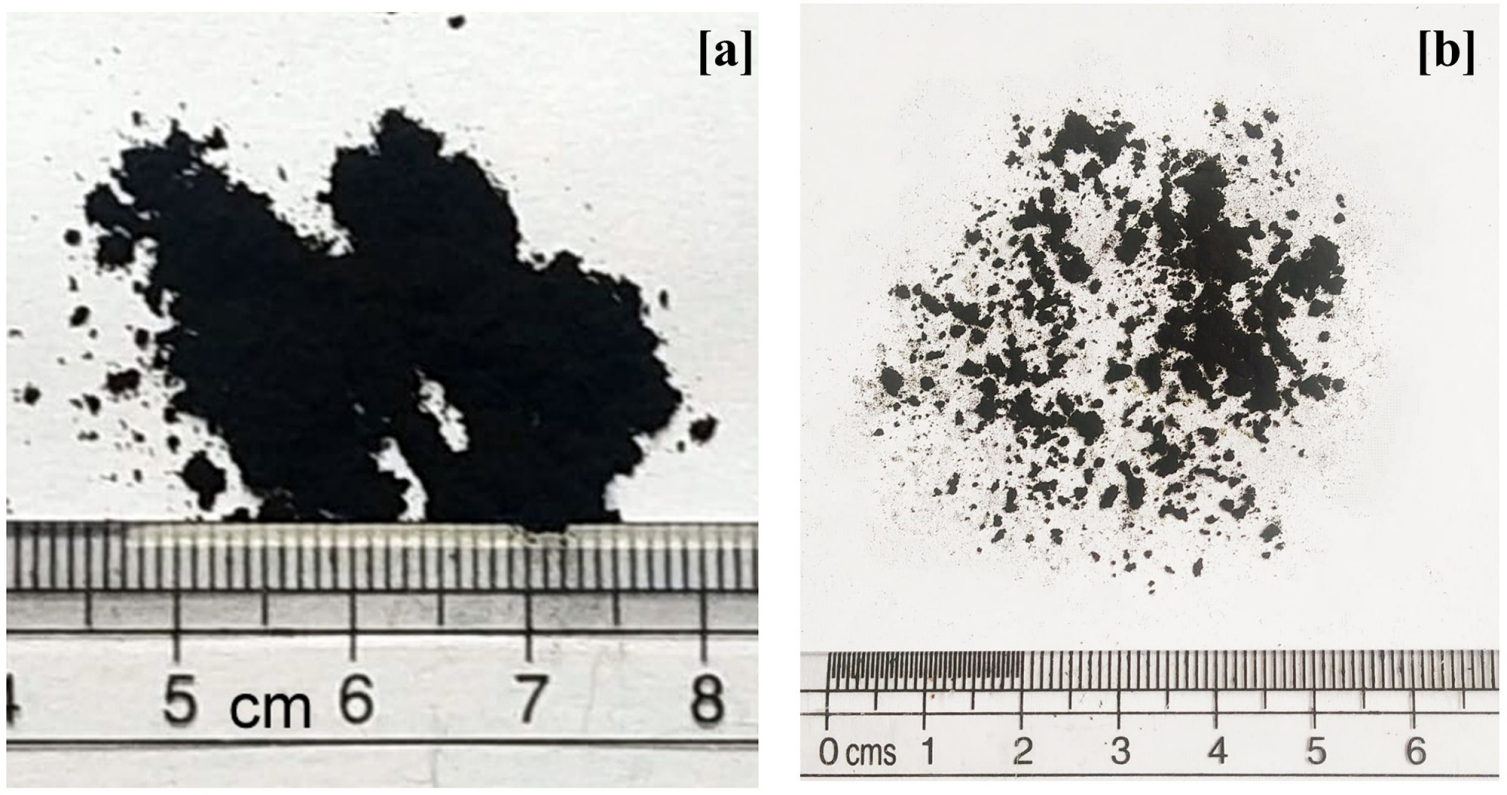
The physical appearance of (a) TPC (b) PPC.

Viscosity grade 30 (VG30) asphalt binder was used as the base (control) binder in this study. The basic properties of the VG30 binder are enlisted in Table 1, along with their requirements as per specifications followed in India. TPC and PPC were respectively obtained from the industrial scale pyrolysis of waste tires and waste plastics and were supplied by Innova Engineering and Fabrication (Mumbai, India). The pyrolytic chars were sieved on a 75 μm (No. 200) sieve and the material passing the sieve was used for asphalt modification. Fig 2 shows the physical appearance of TPC and PPC.

**Table 1:** Properties of base bitumen (VG30).

Property	Requirements*	Results
Absolute viscosity, 60°C, poise	2400–3600	3410
Kinematic viscosity, 135°C, cSt	min 350	525
Penetration, 25°C, 100 g, 5 s, 0.1 mm	min 45	51.6
Softening point (R&B), °C	min 47	52.7
Flash point (COC), °C	min 220	280
Solubility in trichloroethylene, %	min 99	>99
***Properties of RTFO residue***		
Viscosity ratio, 60°C	max 4	1.15
Ductility, 25°C, cm	min 40	>100

*Requirements as per IS: 73 [22], Indian Standard on ‘Paving Bitumen Specification’.

### Preparation of modified asphalt binders

TPC and PPC modified binders were prepared using a high shear mixer equipped with rotor-stator assembly. The control (VG30) asphalt binder was heated to 160°C followed by gradual addition of 10% by weight of TPC and PPC particles. High shear mixing was continued for 30 min at a shear rate of 12,000 rpm to obtain the modified binders. The abbreviations ’TPCMA’ and ’PPCMA’ are used to denote TPC-modified asphalt and PPC-modified asphalt.

### Aging methods

**Fig 3:**
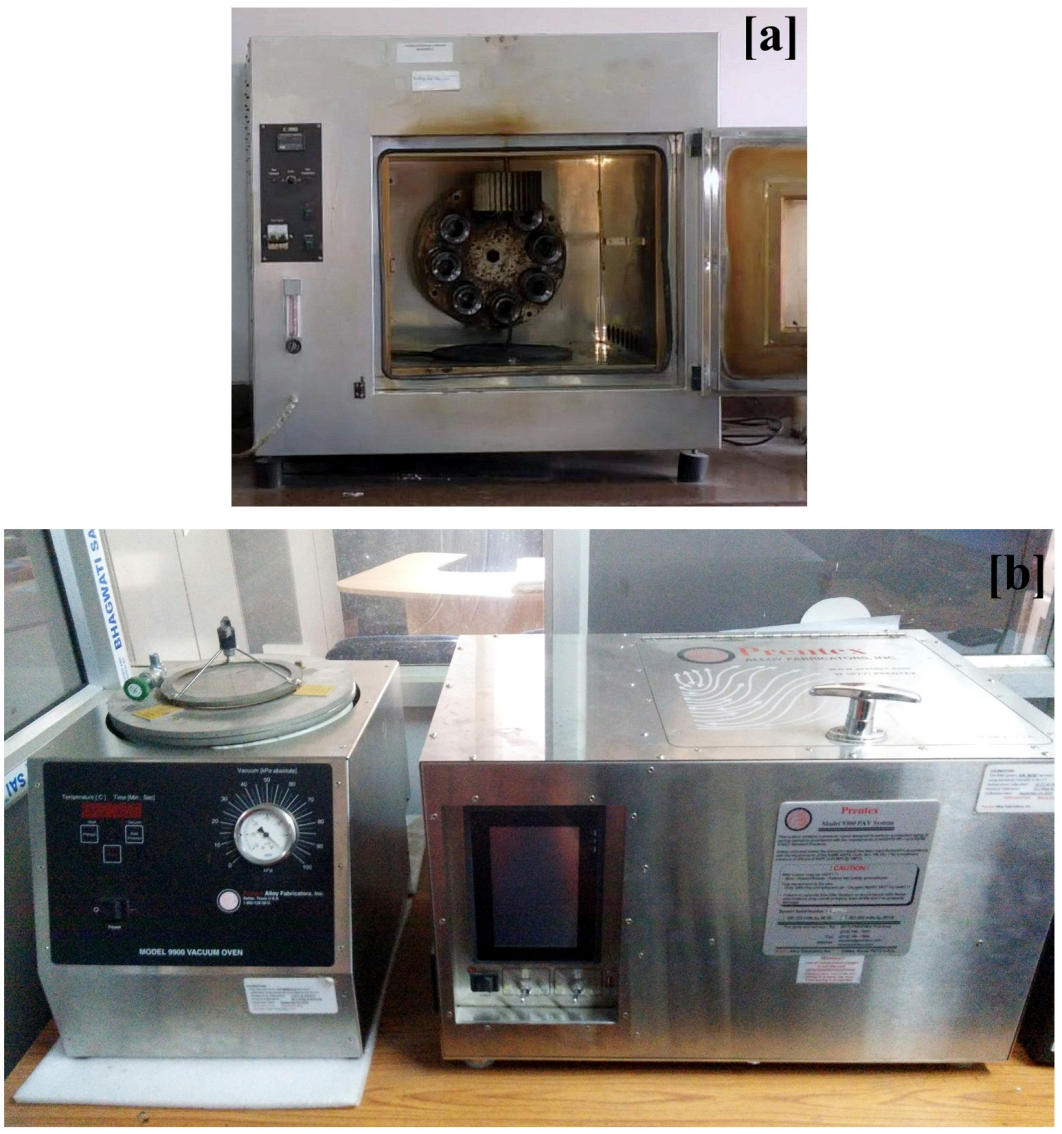
(a) RTFO device used for simulation of short-term aging, and (b) PAV device used for simulation of long-term aging.

Short-term aged binders were obtained on a rolling thin film oven (RTFO) operating at 163°C for 85 min and airflow of 4000 mL/min according to ASTM D2872-19 [23]. Binders were then simulated for long-term aging on a pressure aging vessel (PAV) according to ASTM D6521-19 [24]. The aging temperature was 100°C with air pressure maintained at 2.1 MPa for 20 h. All binder samples were then vacuum degassed at 170°C under an absolute pressure of 15.0 kPa. PAV aging of binders was preceded by RTFO aging. Fig 3 shows the photographs of RTFO and PAV used in this study.

### Rheological tests

Rheological properties of the asphalt binders were measured on a DSR (make: Anton Paar MCR-102). Frequency sweeps were conducted in three decades (0.1–1, 1–10, 10–100 rad/s) at 60°C at low strain (to measure the binder properties in the linear viscoelastic domain). Complex shear modulus (G*) and phase angle (δ) were determined at different frequencies. Based on complex viscosity data derived from oscillatory shear frequency sweep tests, the ZSV of the binders was determined using the Cross-model (Eq 1):

$$\eta *=\frac{\eta_{0}-\eta_{\infty }}{1+{\left(k\omega \right)}^{m}}+\eta_{\infty }$$ (1)

where, η* = complex viscosity (Pa.s); *η*_*0*_ = ZSV (Pa.s); *η*_*∞*_ = viscosity at infinite frequency; *ω* = angular frequency (rad/s), *k* and *m* are model constants.

MSCR test was conducted at 60°C to investigate the high-temperature rutting resistance of the binders under different aging states. Two stress levels (0.1, 3.2) were used with 1 s creep and 9 s recovery intervals. A total of 20 cycles were used at 0.1 kPa (10 cycles for conditioning and next 10 cycles for data acquisition), and 10 cycles were used at 3.2 kPa stress levels as per ASTM D7405-20 [25]. Nonrecoverable creep compliance (*J*_*nr*_) was calculated using Eq 2:

$$J_{nr}=\frac{\varepsilon_{nr}}{\sigma }$$ (2)

where, *ε*_*nr*_ = non-recovered strain and *σ =* stress level.

The BYET is based on the notion that crack propagation in the asphalt pavement is a function of an energy threshold where the applied stress is greater than the binder’s resistance to damage [26,27]. In the BYET, the asphalt binder’s resistance to yield failure under a monotonic loading (at a fixed strain rate) is assessed. To evaluate the binder’s yield energy, AASHTO TP 123–16 [28] specifies the application of a total 4167% strain achieved by rendering a fixed strain rate of 2.315% s^–1^ for 30 min. The test was carried out at 15 and 25°C (representing usual intermediate service temperatures) on long-term aged binders. During the test, the asphalt binder starts to yield after reaching a peak point, implying that the binders cannot resist further stress. The area up to maximum shear stress from the stress-strain curve gives the binder yield energy. All rheological tests were performed thrice, and the average results were reported.

### Rheological aging indices

To quantitatively characterize the effect of aging, four aging indices (AI) based on rheological properties were formulated, as shown in Eqs 3–6. These indices are based on binder complex modulus (G*), phase angle (δ), ZSV, and MSCR *J*_*nr*_ before and after being subjected to aging. Rheological aging indices defined as the ratio of a parameter before and after aging have been used in several asphalt aging studies [29,30]. The rheological indices are defined such that a lower value of each aging index represents more minor changes due to aging in the rheological property under consideration and, therefore, a lower aging susceptibility. This definition allows convenience to interpret the rheological as well as FTIR-based indices.


$$AI(G*)=\frac{G*\text{in}\;\text{aged}\;\text{state}}{G*\text{in}\;\text{unaged}\;\text{state}}$$ (3)

$$AI(\delta )=\frac{\delta \;\text{in}\;\text{unaged}\;\text{state}}{\delta \;\text{in}\;\text{aged}\;\text{state}}$$ (4)

$$AI(ZSV)=\frac{ZSV\;\text{in}\;\text{aged}\;\text{state}}{ZSV\;\text{in}\;\text{unaged}\;\text{state}}$$ (5)

$$AI(J_{nr})=\frac{J_{nr}\;\text{in}\;\text{unaged}\;\text{state}}{J_{nr}\;\text{in}\;\text{aged}\;\text{state}}$$ (6)

### Spectroscopic analyses

FTIR spectroscopy was done to identify and quantify functional groups for the aging characterization of the TPC and PPC-modified asphalt binders. A PerkinElmer UATR Two FTIR spectrometer working in attenuated total reflection (ATR) mode was used with an accumulation of 64 scans in the 400–4000 cm^–1^ range. Binder samples were dissolved in tetrahydrofuran at a concentration of 10% w/v. ^1^H NMR analysis was conducted on a Bruker 400 MHz NMR. The binder samples (about 20 mg) were dissolved in deuterated chloroform (CDCl_3_) and placed in 5 mm NMR tubes. FTIR and NMR were performed twice for each binder, and average results were reported.

## Results and discussion

### Frequency sweep

**Fig 4:**
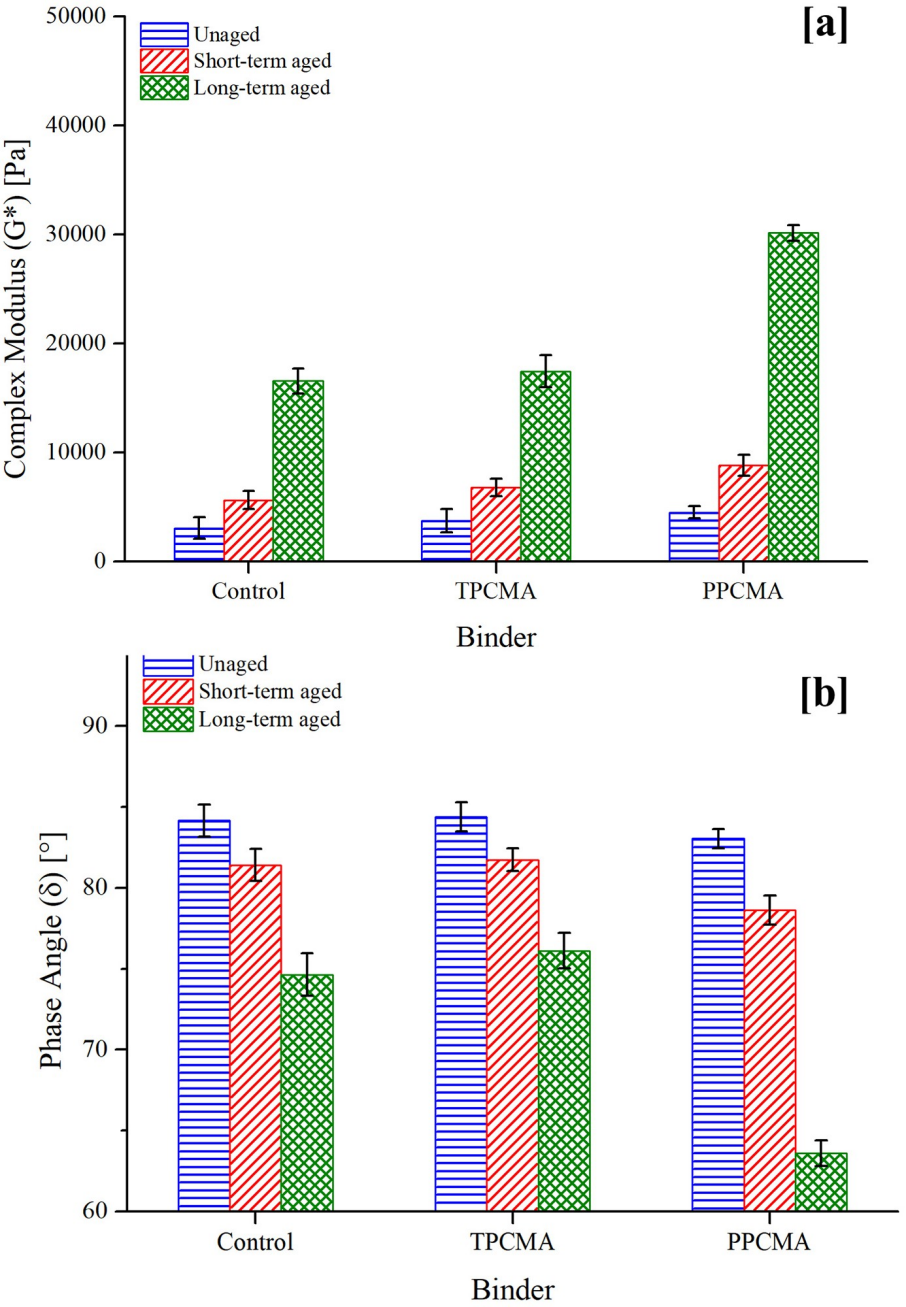
Frequency sweep result at 10 rad/s (a) G* (b) δ.

The results of two viscoelastic rheological parameters G* and δ measured at 60°C for PPC and TPC modified asphalt (abbreviated as PPCMA and TPCMA) are presented in Fig 3 at 10 rad/s frequency. The error bars represent one standard deviation from the replicate measurements. Similar trends were found at other frequencies. The G* describes the total resistance to deformation of a binder when subjected to a repeated sinusoidal shear load, whereas δ reflects the ratio of elastic and viscous behavior of the binder. A higher G* and a lower δ are desirable attributes for an improved high-service temperature performance [31,32]. It can be seen from Fig 4 that the addition of both TPC and PPC leads to an increased G*, indicating that modification by both pyrolytic chars improves the stiffness of the binder. However, an appreciably lower δ is only seen for the PPC-modified binder. As expected, aging causes an upward shift in the G* values of the control and modified binders. Aging causes a stiffer (higher G*) and more elastic (lower δ) response of the asphalt binder. Results indicate that both TPC and PPC have a positive effect on the deformation resistance of the binder. In previous studies, the addition of tire and plastic pyrolytic char to the asphalt binder resulted in a similar enhancement in complex modulus [16,19,20].

**Fig 5:**
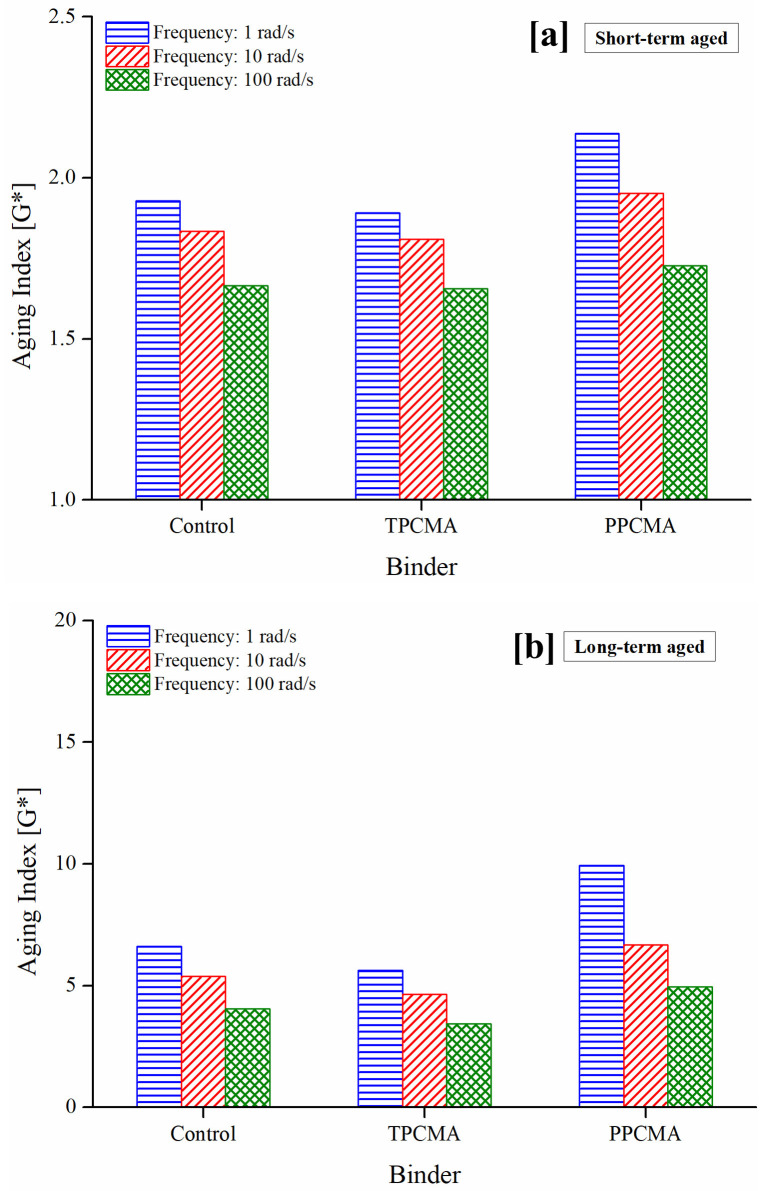
Aging index based on G* under (a) short-term aging, and (b) long-term aging.

**Fig 6:**
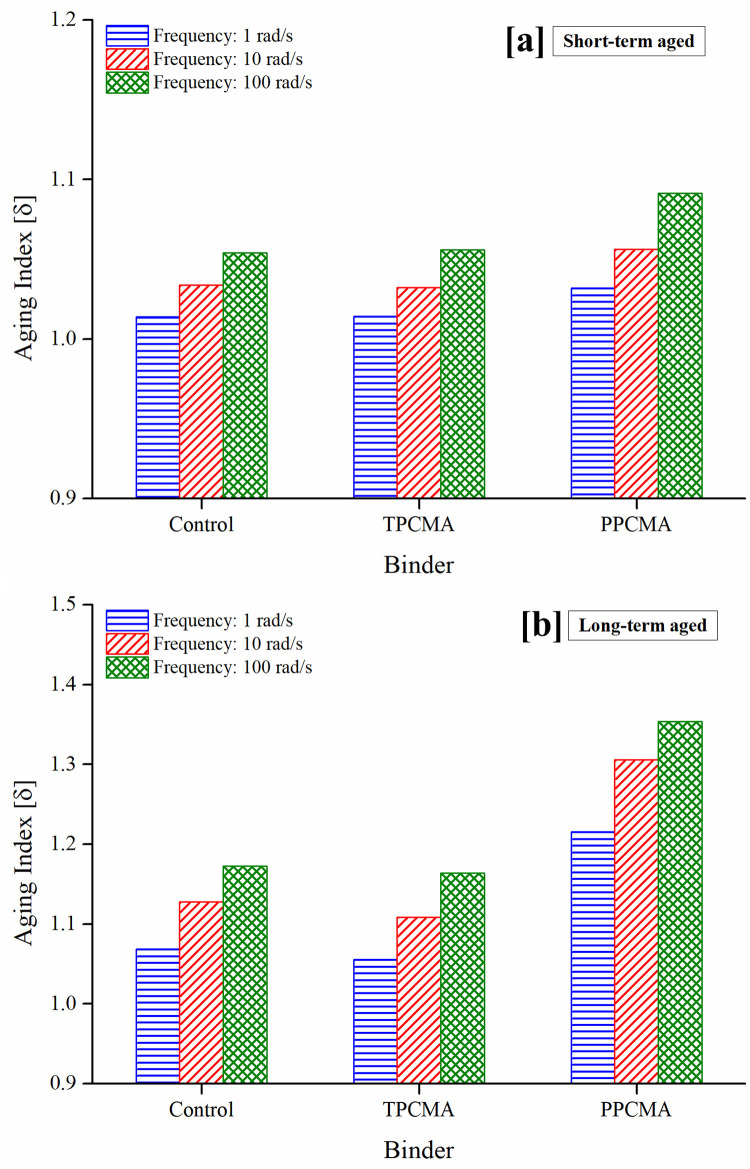
Aging index based on δ under (a) RTFO and (b) PAV.

The aging indices derived from G* and δ are shown in Figs 5 and 6, respectively, at three frequencies (1, 10, and 100 rad/s). As noted earlier, a lower value of the indices indicates lower changes in the material properties on aging. Figs 5 and 6 show that the indices are the lowest when TPC is used under both short- and long-term aged states and the three frequencies. It is therefore inferred that the aging susceptibility of the TPC-modified binder is the least. Lower frequencies lead to higher aging index values, suggesting that aging is more severe at low frequencies (representing slower traffic speeds). A discussion on mechanisms contributing to the observed trends is provided later in this section.

### Zero shear viscosity (ZSV)

**Fig 7:**
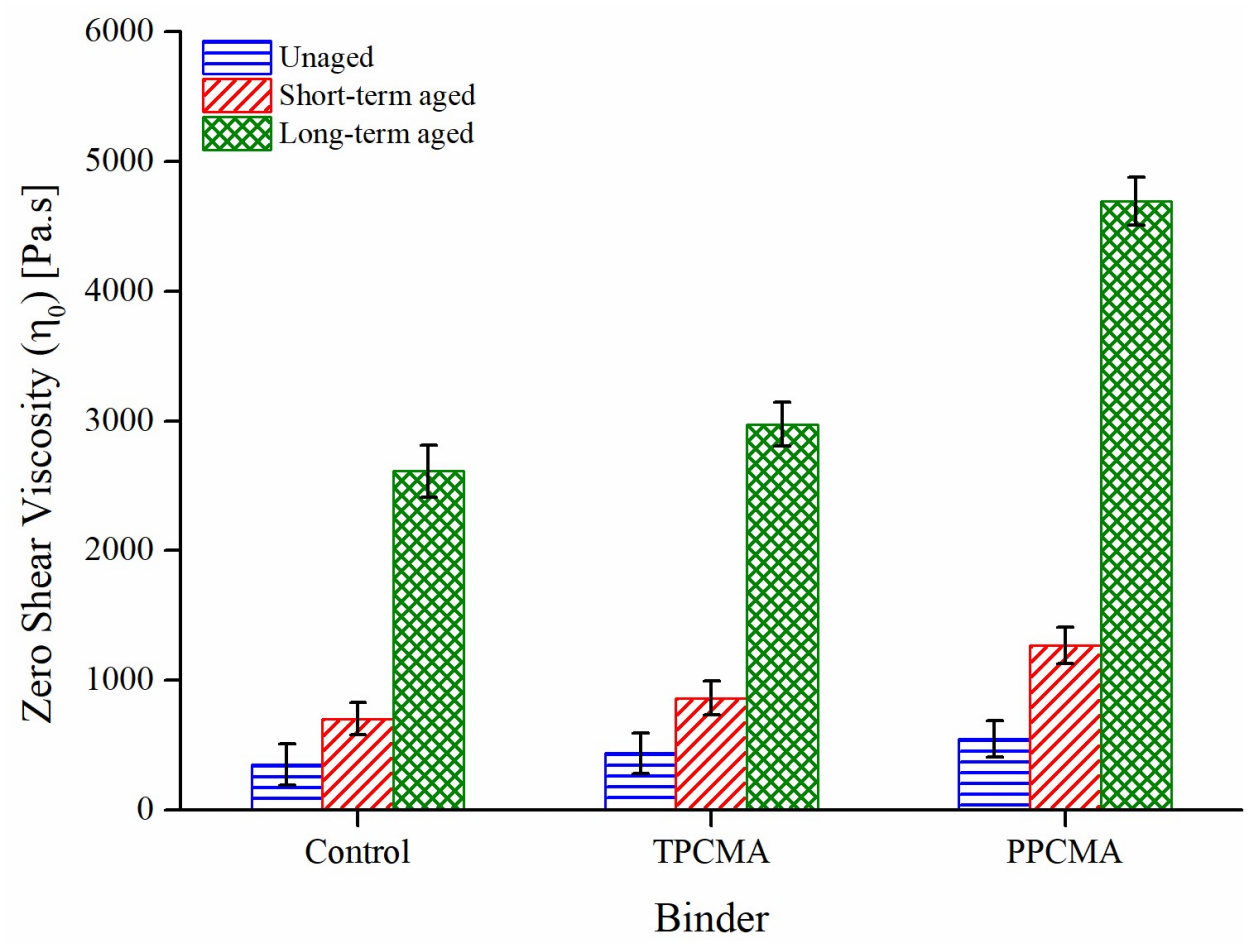
ZSV results at 60°C.

ZSV indicates the viscosity of an asphalt binder under a very low shear rate (or angular frequency) and is often used as a parameter to evaluate high-service temperature properties of modified binders since the response of these binders may not follow a Newtonian relationship at high-service temperatures. From Fig 7, it is seen that the ZSV of the asphalt binders enhances with an increment in the aging severity/period. A sharp increase in ZSV occurs from short-term to long-term aged conditions. The ZSV values of TPC and PPC-modified binders are larger than the control binder under the three aging states, indicating that TPC and PPC can improve the high-temperature performance of asphalt. This finding is consistent with the previous discussion based on G*.

**Fig 8:**
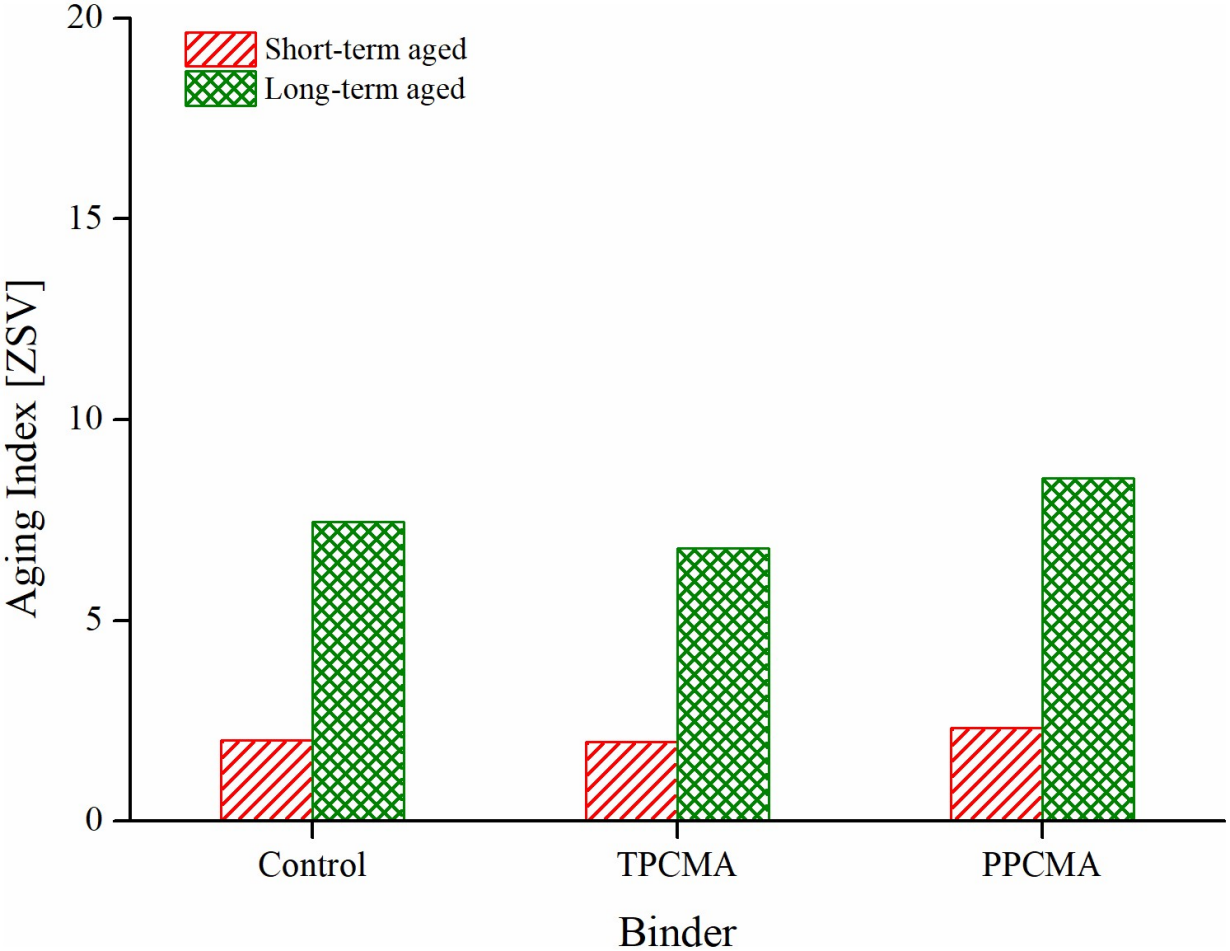
Aging index based on ZSV.

The aging index formulated based on ZSV is computed as the ratio of ZSV after and before aging, and therefore a lower value indicates fewer changes in ZSV due to aging. Fig 8 presents the result of the ZSV-based aging index in short-term and long-term aging conditions. The long-term aging index is significantly higher than the short-term index. This is due to the higher degree of aging undergone by the binder in the PAV process. Under both aging conditions, the aging index for TPC-modified binders is found to be the lowest. The G* and δ based aging indices also ranked the binders in the same order.

### Multiple stress creep and recovery (MSCR)

**Fig 9:**
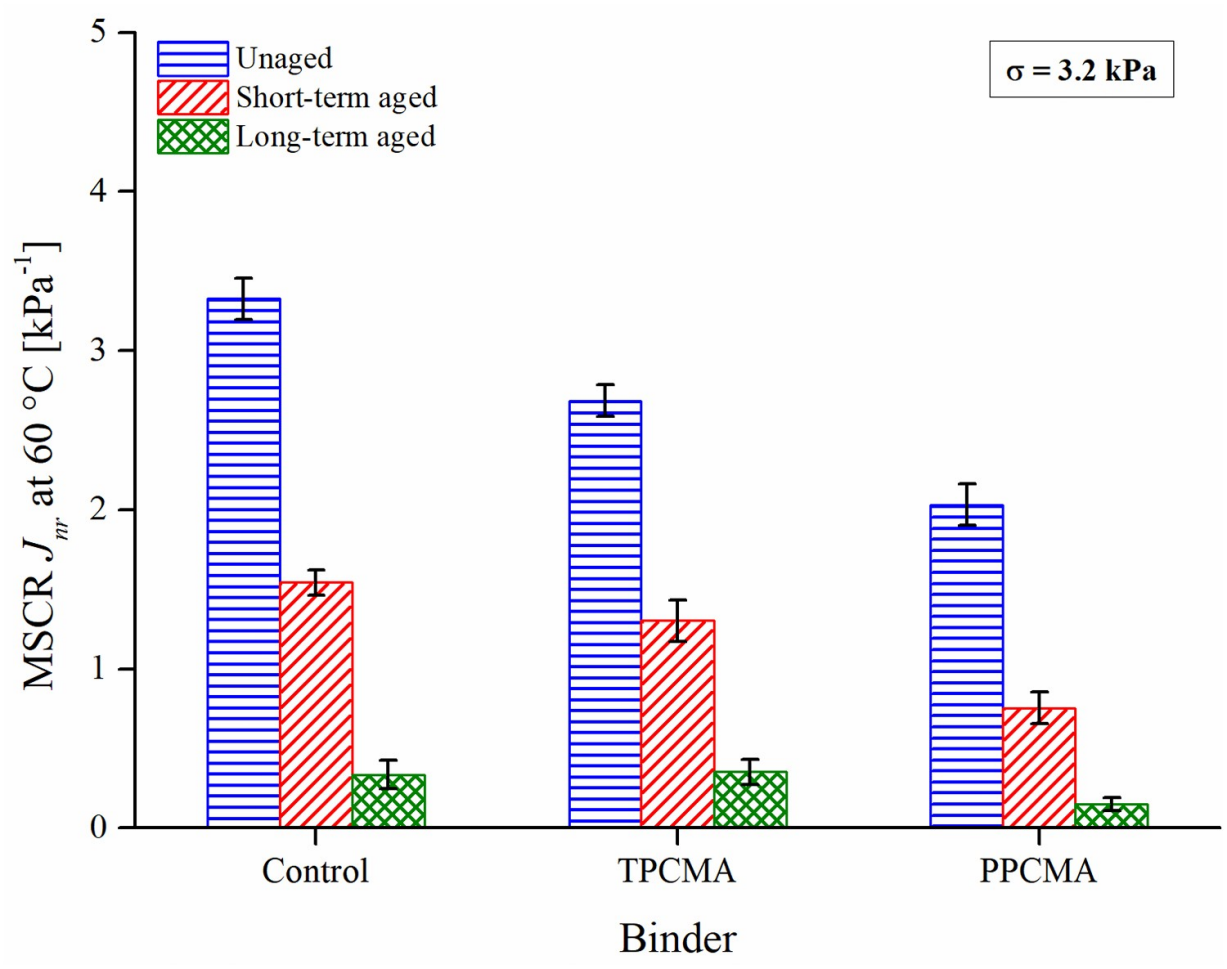
MSCR *J*_*nr*_ result at 60°C.

**Fig 10:**
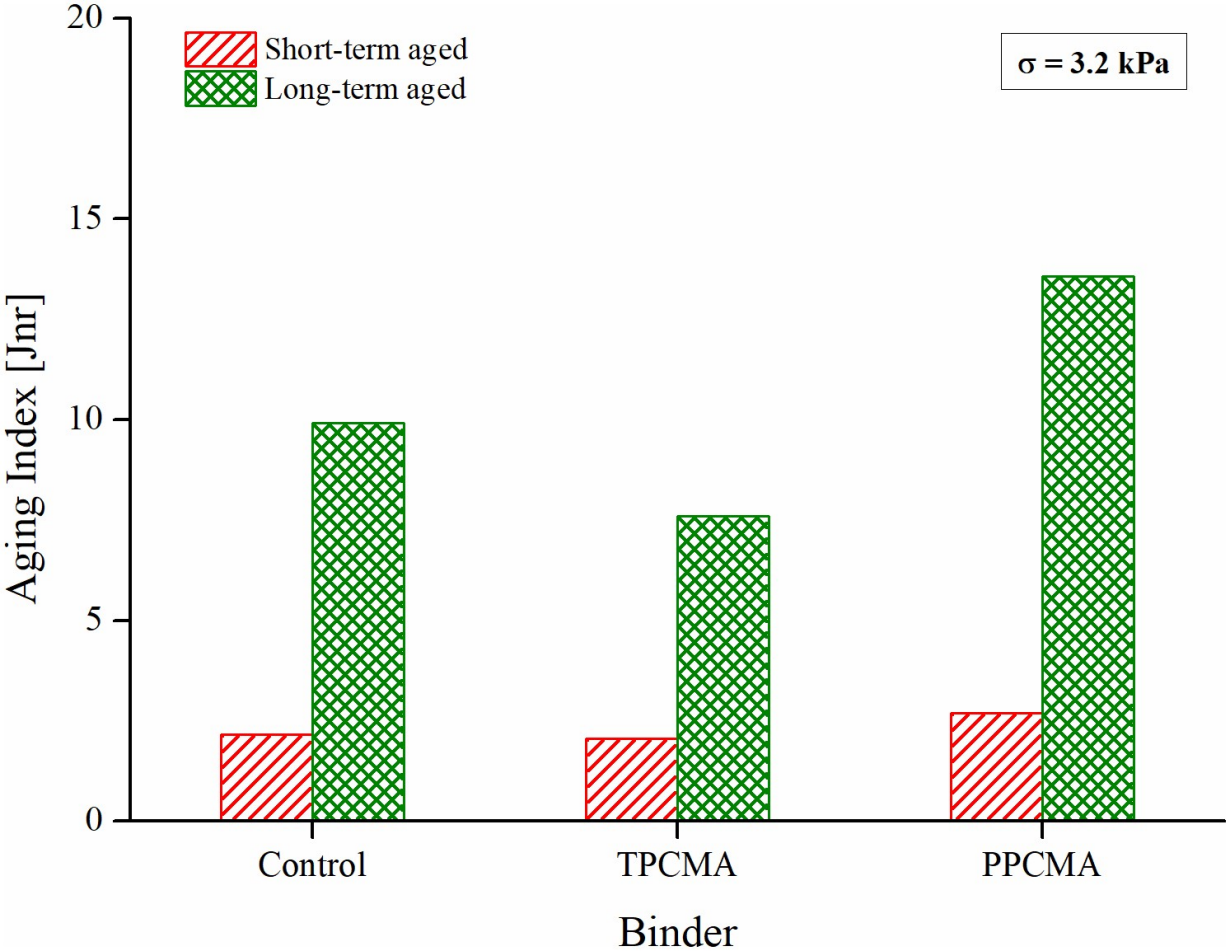
Aging index based on MSCR *J*_*nr*_.

The nonrecoverable creep compliance (*J*_*nr*_) from the MSCR test is commonly used to assess the high-temperature rutting performance of asphalt binders. Smaller the value of *J*_*nr*_, the better the high-temperature resistance of the binder since it indicates lower unrecovered strain at the end of an MSCR creep-recovery cycle. Fig 9 shows that aging has a significant effect on the *J*_*nr*_ of binders. *J*_*nr*_ decreases with a higher degree of aging with a minimum value for the long-term aged binders. Both TPC and PPC reduce the *J*_*nr*_ values at the three aging conditions compared to the base binder indicating the superior rutting resistance derived from the addition of the pyrolytic chars. These results correspond well with the earlier results of ZSV and G* of the binders. A similar reduction in MSCR *J*_*nr*_ was also observed in previous studies with incorporation of pyrolytic char in asphalt binder [18,20]. The aging index based on *J*_*nr*_ shown in Fig 10 ranks the binders in the same order as for the indices based on G*, δ, and ZSV. The lowest index is observed for the TPC-modified binder, followed by the control and the PPC-modified binder. Now, a discussion is presented to explain the observed trends in the aging performance of the binders.

### Discussion on contributing mechanisms

Carbon black is added to tire rubber during its manufacturing to improve its strength and aid in resistance to abrasion. TPC, therefore, consists of the recovered carbon black, inorganic compounds used in tire making, and condensed by-products formed during the pyrolysis [17,33,34]. Carbon black has been reported to function as an effective antioxidant to the asphalt binders due to chemical species such as quinones, carboxyphenols, phenols, and lactones [35]. When subjected to aging, these chemical species may have a higher tendency to react with oxygen than the asphalt binder, leading to a better aging resistance of the TPC modified binder. Wang et al. [18] used aging index based on MSCR *J*_*nr*_ and also reported improvements in binder aging resistance with tire pyrolytic carbon black and attributed them to the presence of functional groups which are more likely to react with oxygen than bitumen.

Another possible mechanism for better aging resistance for TPC modified binder was reported by Wang et al. [18] based on the results of electron spectroscopy for chemical analysis (ESCA), infrared, and ultraviolet spectroscopic studies on TPC modified bitumen by Chaala et al. [36]. The TPC particles absorb maltenes when added to bitumen, and a thin boundary layer composed of asphaltenes exists on the TPC surface. Now, the aging of bitumen is mainly related to the depletion of maltenes and the formation of more asphaltene-like compounds. When the binder is aged, such a system (TPC particle surrounded by asphaltenes) inhibits the depletion of maltenes and thus contributes to improving the binder aging resistance. However, such in-depth chemical studies on PPC modified asphalt are currently not available.

Char obtained from plastic waste pyrolysis is reported to have higher amounts of volatile matter [37,38]. Proximate analysis conducted on TPC and PPC revealed that volatile matter content in PPC was 27.7% compared to ~4% in TPC. The volatile matter becomes gaseous and will likely escape when the binder undergoes aging at high temperatures (short-term aging) or under a combination of high temperature and high pressure (long-term aging). This, in turn, is expected to contribute to a higher aging index of the PPC-modified asphalt binders.

### Binder yield energy test (BYET)

An asphalt binder should perform well against rutting at high-service temperatures and fatigue cracking damage at intermediate service temperatures. The susceptibility of long-term aged TPC and PPC modified binders to fatigue damage was characterized using the BYET. Based on the stress-strain response, the area under the curve up to the peak stress is obtained as the binder yield energy. Higher yield energy corresponds to a better fatigue damage resistance. The BYET was performed on long-term aged binders at two intermediate service temperatures: 15 and 25°C.

**Fig 11:**
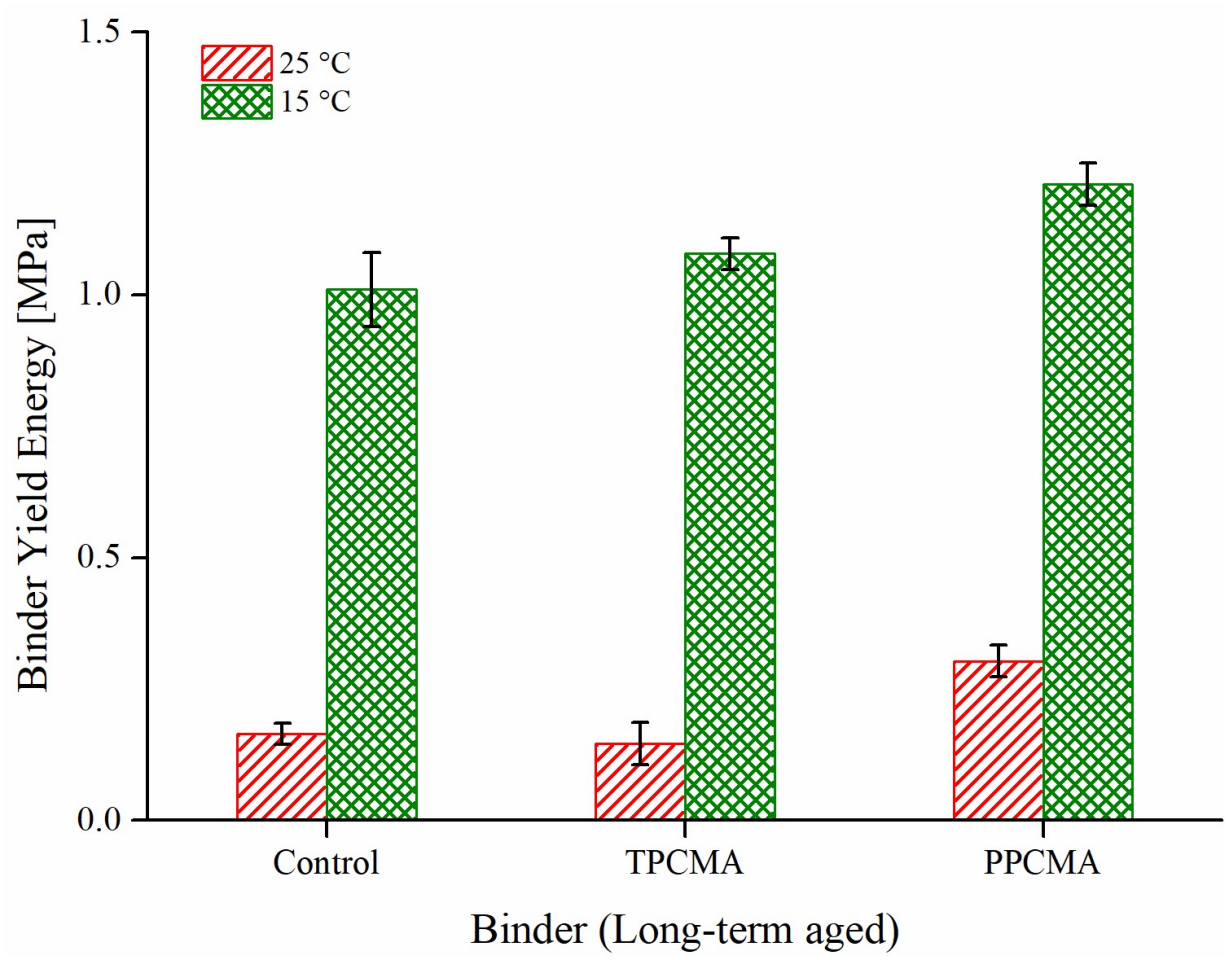
BYET results at 15 and 25°C.

Despite showing higher aging indices, the PPC modified binder demonstrates the highest yield energy at both temperatures (Fig 11). On the other hand, the control and TPC-modified binders have quite close values of the yield energy at both test temperatures. It is to be noted that the aging evaluation based on rheological variables compares the stiffness properties (G*, ZSV, *J*_*nr*_) of the binders before and after aging. Although binder stiffness increased with the addition of PPC, this is accompanied by an improved elasticity as evidenced by a lower phase angle of aged PPC-modified asphalt than the control binder, which can impede the damage generation and propagation in the BYET test. However, the improved fatigue resistance needs to be confirmed further by testing the asphalt mixtures fabricated with the modified binders.

### FTIR spectroscopy

**Fig 12:**
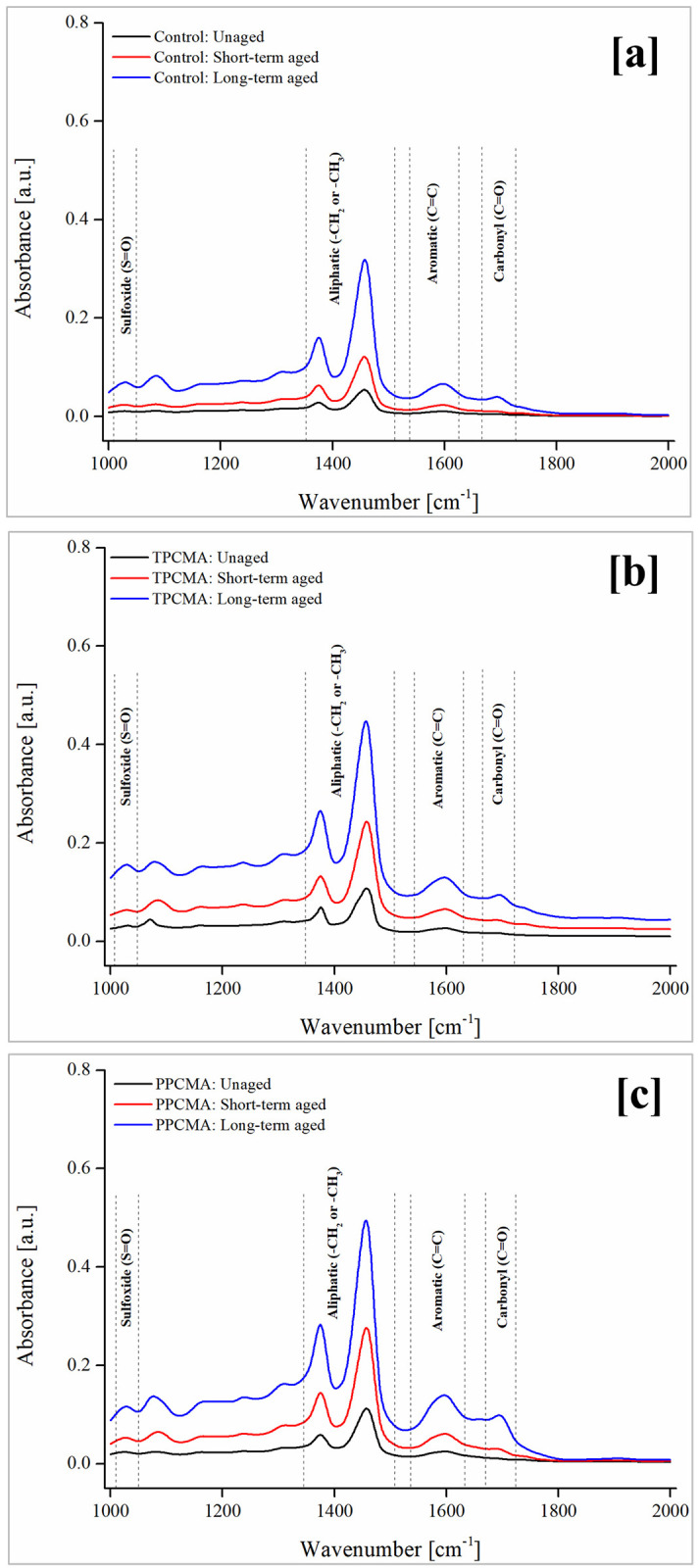
FTIR spectra under different aging state (a) Control (b) TPCMA (c) PPCMA.

Chemical composition of asphalt binder changes with aging. During oxidative aging, the chemical groups in the asphalt binder react with oxygen, and therefore monitoring the changes in oxygen-based functional groups is quite helpful to understand changes brought in the binder due to aging. FTIR was used to observe the progress of chemical functionalities for control, TPC-modified and PPC-modified binders subjected to short- and long-term aging. FTIR spectra of the binders under different aging states are shown in Fig 12. Peaks corresponding to two oxygenated functions, namely carbonyl (C = O, centered around 1700 cm^–1^) and sulfoxide (S = O, centered around 1030 cm^–1^), are used to evaluate changes caused due to aging in asphalt binders [39–41]. Distinct regions corresponding to carbonyl and sulfoxide are also indicated in Fig 12. It can be seen that new carbonyl and sulfoxide functional groups are formed as the duration of aging increases for all binders. These groups are formed when other chemical bonds such as C–C, and C = C dismantle and react with oxygen or when sulfur-based compounds of the asphalt react with oxygen [39,42].

Quantitative analysis of the FTIR spectra was performed by calculating carbonyl (C = O) index (CI) and sulfoxide (S = O) index (SI) based on the peaks in distinct regions of the spectra [43] as per Eqs 7 and 8:

$$CI=\frac{A_{1678-1725}}{\sum A}$$ (7)

$$SI=\frac{A_{1010-1043}}{\sum A}$$ (8)

where, ∑ *A* = *A*_1010−1043_ + *A*_1350−1510_ + *A*_1535−1625_ + *A*_1678−1725_.

**Fig 13:**
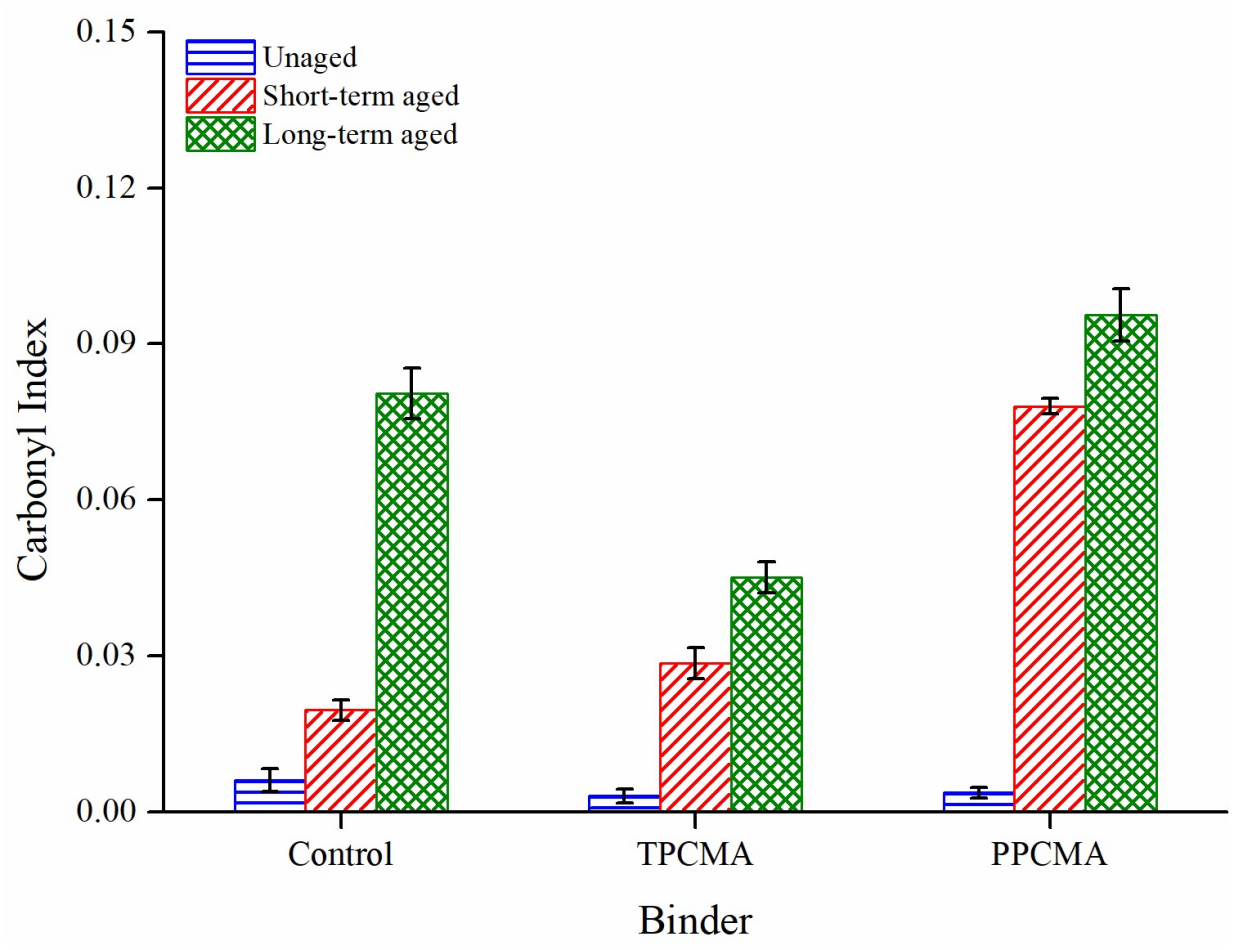
Carbonyl index under different aging states.

**Fig 14:**
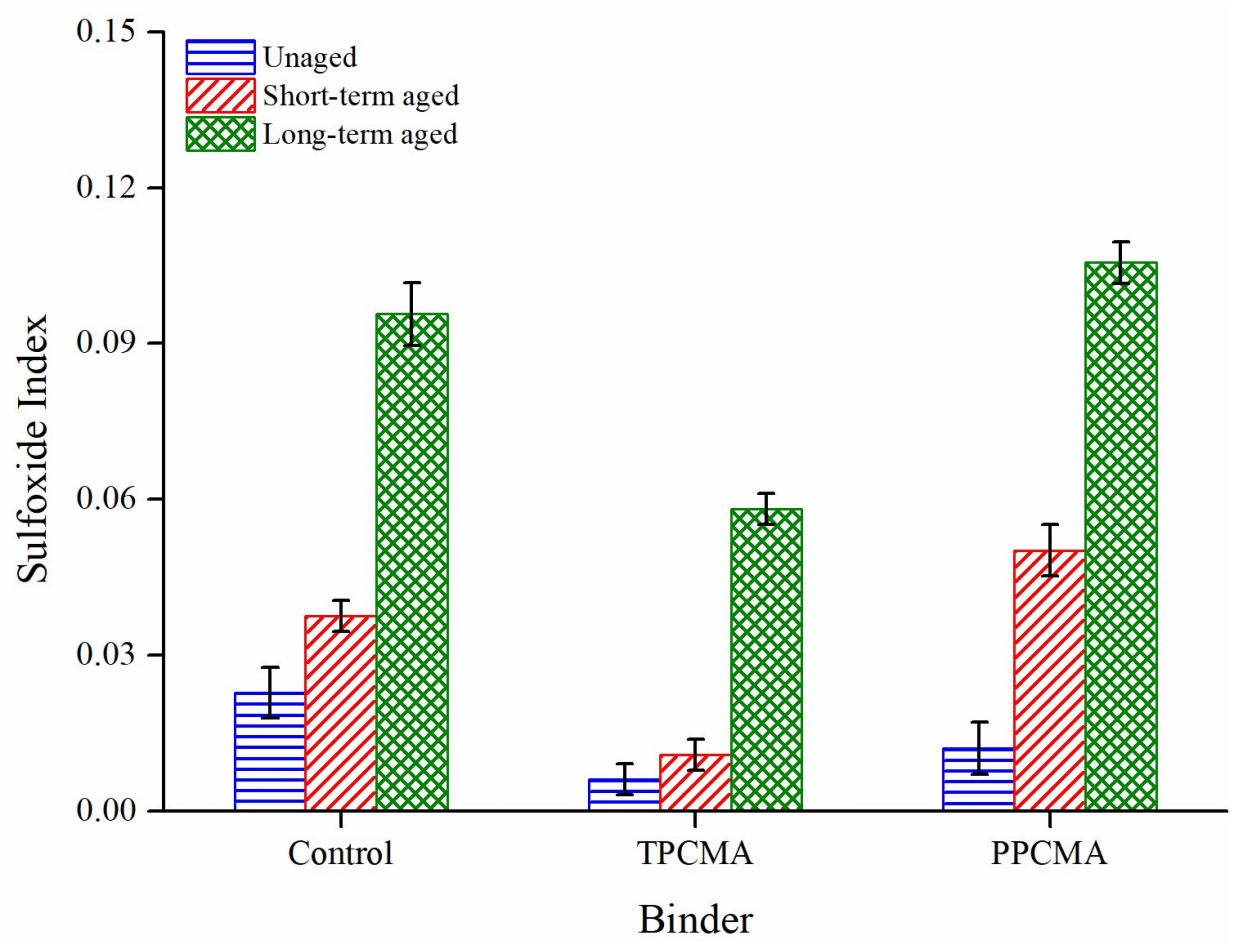
Sulfoxide index under different aging states.

Figs 13 and 14 show the results of FTIR indices for all binders at different aging conditions. In general, all indices consistently increase as the severity of aging progresses in the order: unaged < short-term aged < long-term aged. The highest increase is found between the short-term and long-term aged conditions for all binders. In the short- and long-term aged conditions, the CI and SI are the lowest for the TPC-modified binder. This indicates that the modification by TPC leads to the formation of fewer carbonyl and sulfoxide groups. Therefore, it is inferred that the addition of TPC can help inhibit the increase in chemical functionalities and improve the resistance to thermal-oxidative aging. These findings also coincide with the observations from the rheological aging indices.

### ^1^H-NMR spectroscopy

**Fig 15:**
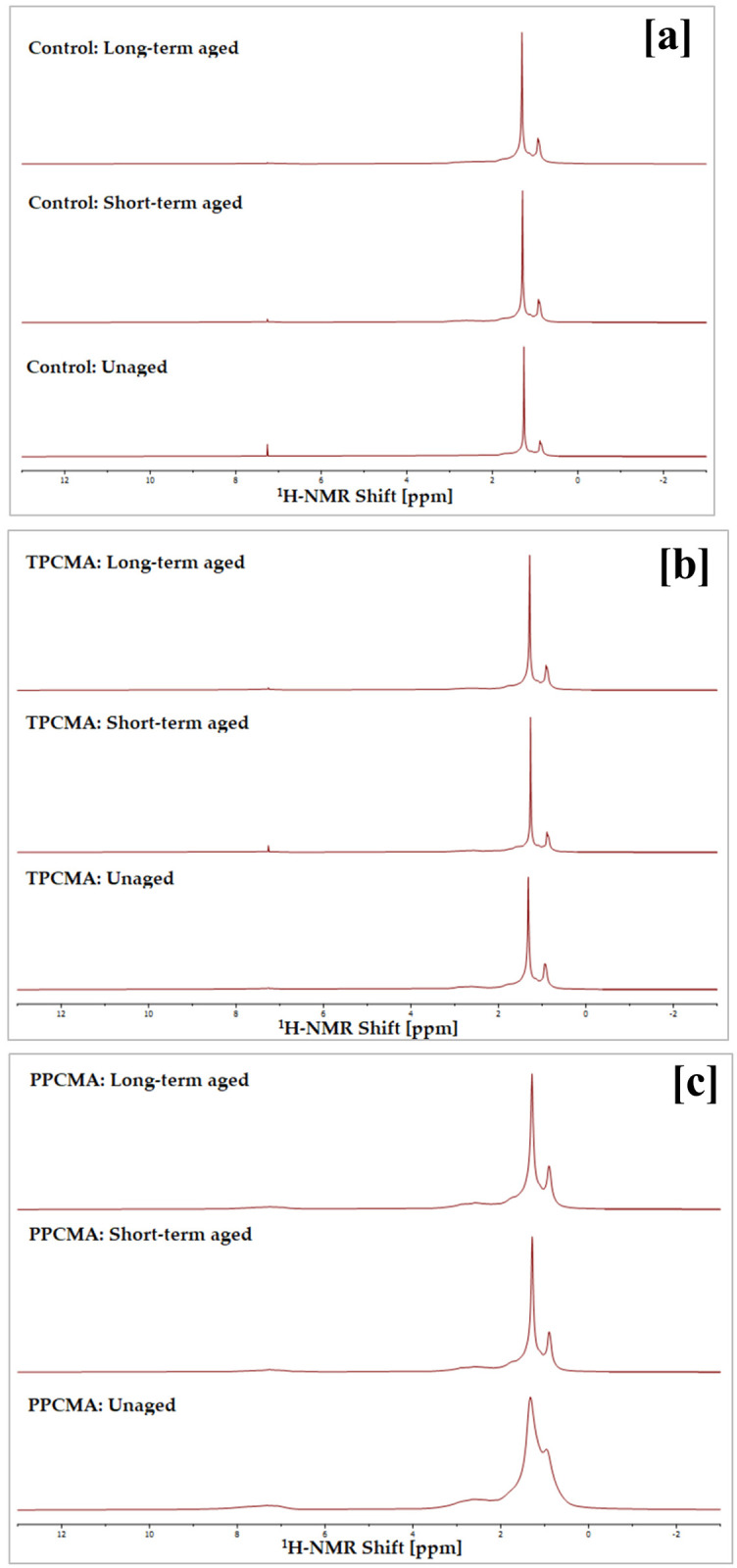
^1^H-NMR spectra under different aging states (a) Control (b) TPCMA (c) PPCMA.

The ^1^H-NMR spectra of control, TPC-modified, and PPC-modified binders in different aging states are displayed in Fig 15. Prominent absorption peaks appear in the region from 0 to 3 ppm and can be assigned to linear and substituted hydrocarbons with saturated alkanes (CH_3_, CH_2_, and CH) [6,44]. The ^1^H spectra of binders at different aging states have relatively similar positions of peaks. For example, the absorption peaks of the control (unaged) binder appear at 0.89, 1.26, and 2.53 ppm, while those for the control (short-term aged) binder appear at 0.92, 1.30, and 2.61 ppm. Due to the possible influence of solvent and magnetic anisotropy, the major peaks of the binders deviate slightly toward high or low shifts. However, no new prominent peaks appear between the ^1^H-NMR spectra of different binders. The two strong peaks around 1.25 and 0.85 ppm are assigned to protons on methyl and methylene groups, respectively [45]. The signal at 7.26 ppm belongs to the solvent (CDCl_3_). No signal on the 4–6 ppm region was observed, which corresponds to olefinic hydrogen, indicating that olefinic hydrocarbons are negligible in the binders. Similar peak assignments for asphalt binders were also reported by Rossi et al. [45].

NMR spectra of a binder in unaged and aged states are reported to be quite similar by other researchers [46], which is also observed in this study. Therefore, a quantitative analysis was performed to understand the differences better. The distribution of protons among aromatic (chemical shift: 6–9 ppm) and aliphatic regions (chemical shift: 0.5–4 ppm) was determined for the three binders at all aging states based on integration and normalization of the ^1^H-NMR spectra. The aliphatic protons were further segregated into three groups based on their positions: H_α_: 2–4 ppm; H_β_: 1–2 ppm; and H_γ_: 0.5–1 ppm. Table 2 presents the details of protons assignment from ^1^H-NMR spectra.

**Table 2:** Proton assignment in ^1^H-NMR [47,48].

Parameter	Chemical shift (ppm)	Type of proton
H_ar_	6.0~9.0	Hydrogen directly linked to aromatic carbon
H_α_	2.0~4.0	Hydrogen linked to α-carbon of aromatic nucleus
H_**γ**_	0.5~1.0	Hydrogen linked to γ-carbon of aromatic nucleus and γ beyond CH_3_, CH group
H_β_	1.0~2.0	Hydrogen linked to β-carbon of aromatic nucleus and β beyond CH_2_, CH group

**Fig 16:**
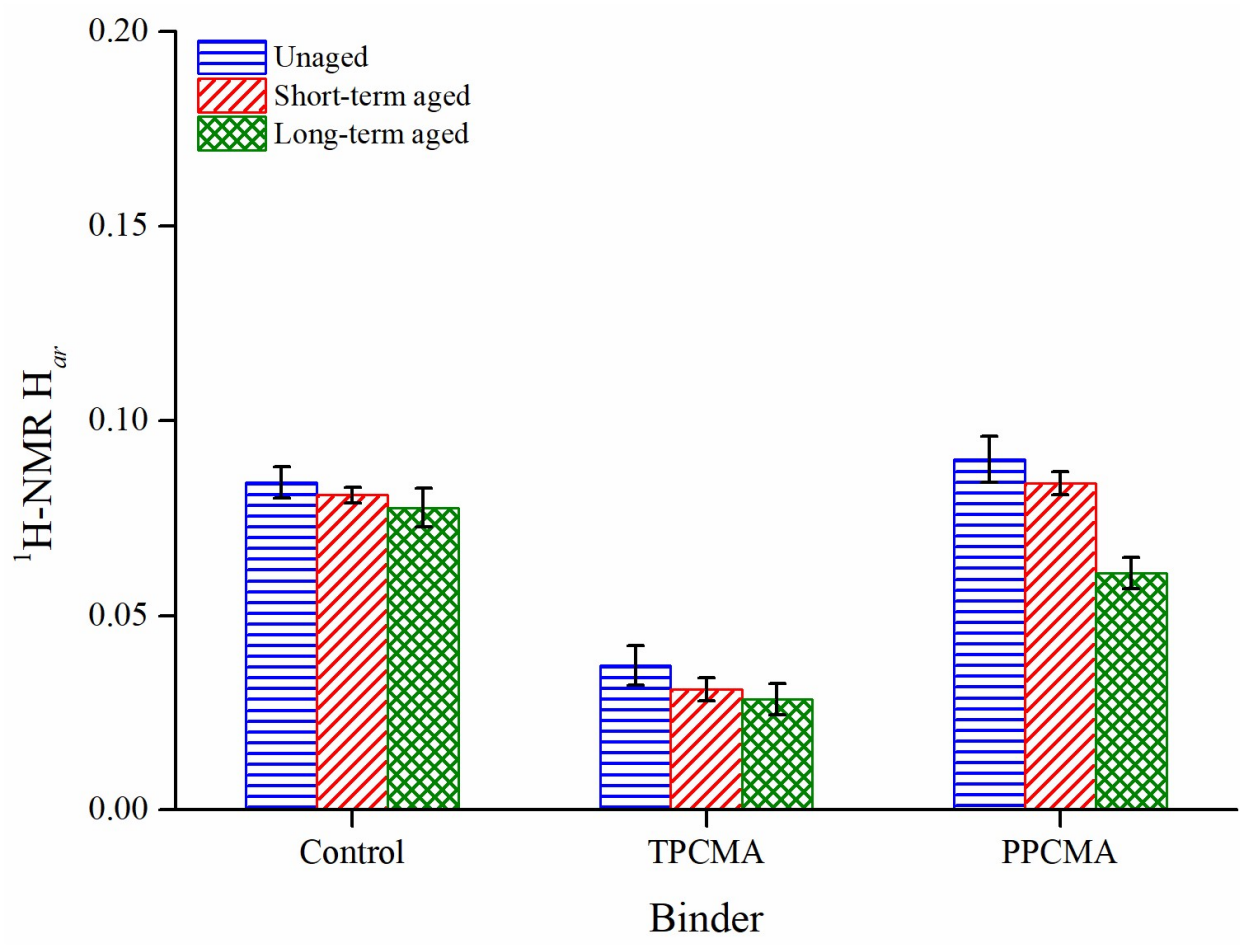
H_ar_ results.

Fig 16 shows the aromatic hydrogen (H_ar_) distribution of all binders in unaged, short-term aged, and long-term aged states. Comparing the H_ar_ values for unaged binders, it can be seen that H_ar_ of TPC-modified asphalt declined whereas that for PPC-modified asphalt slightly increased compared to the control binder. This suggests that some light asphalt components such as aromatics are added to the binder on modification with PPC. Furthermore, the H_ar_ values of aged binders were lower than unaged binders. This may be due to higher condensation and substitution of aromatic structures in the binders due to aging, as also concluded by Ma et al. [47].

**Fig 17:**
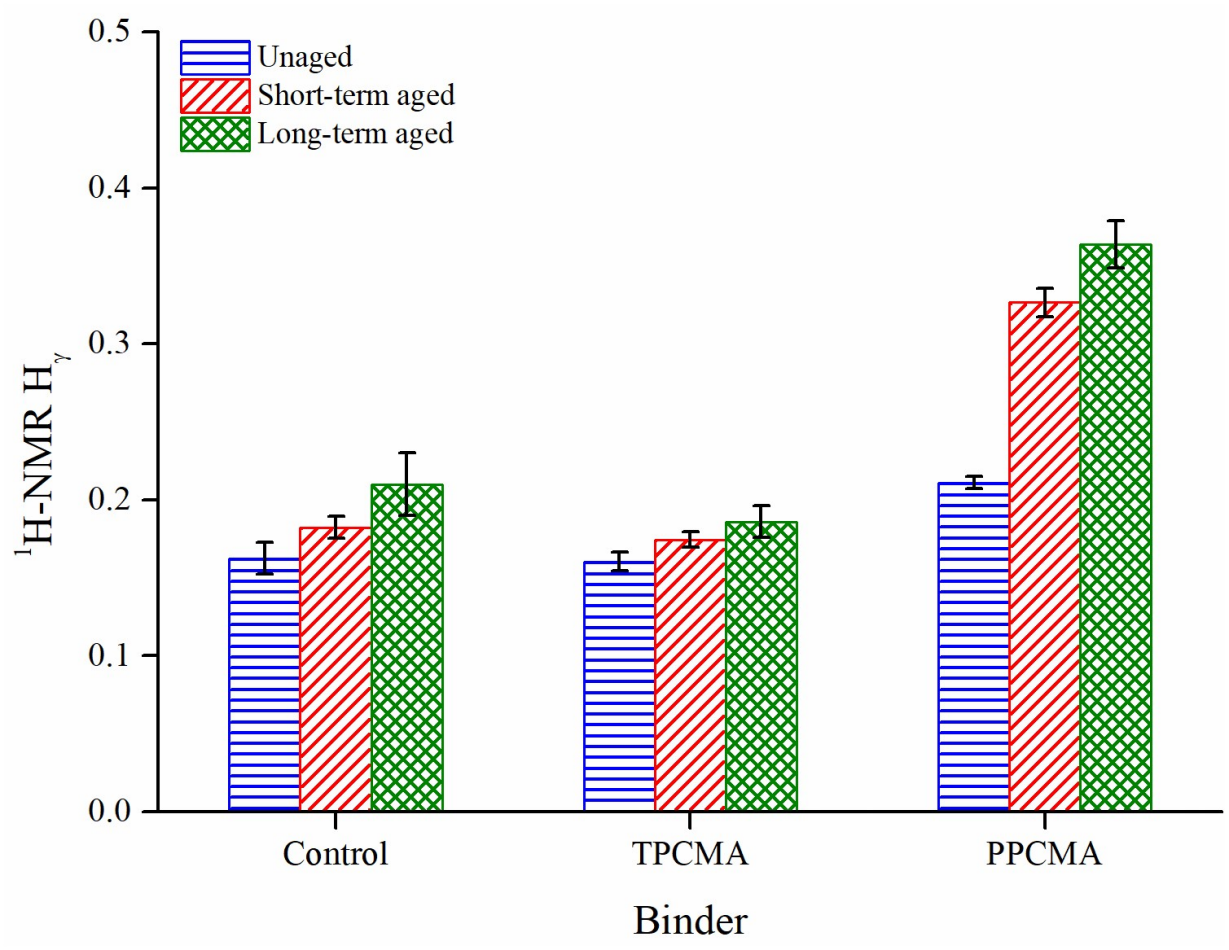
H_γ_ results.

**Fig 18:**
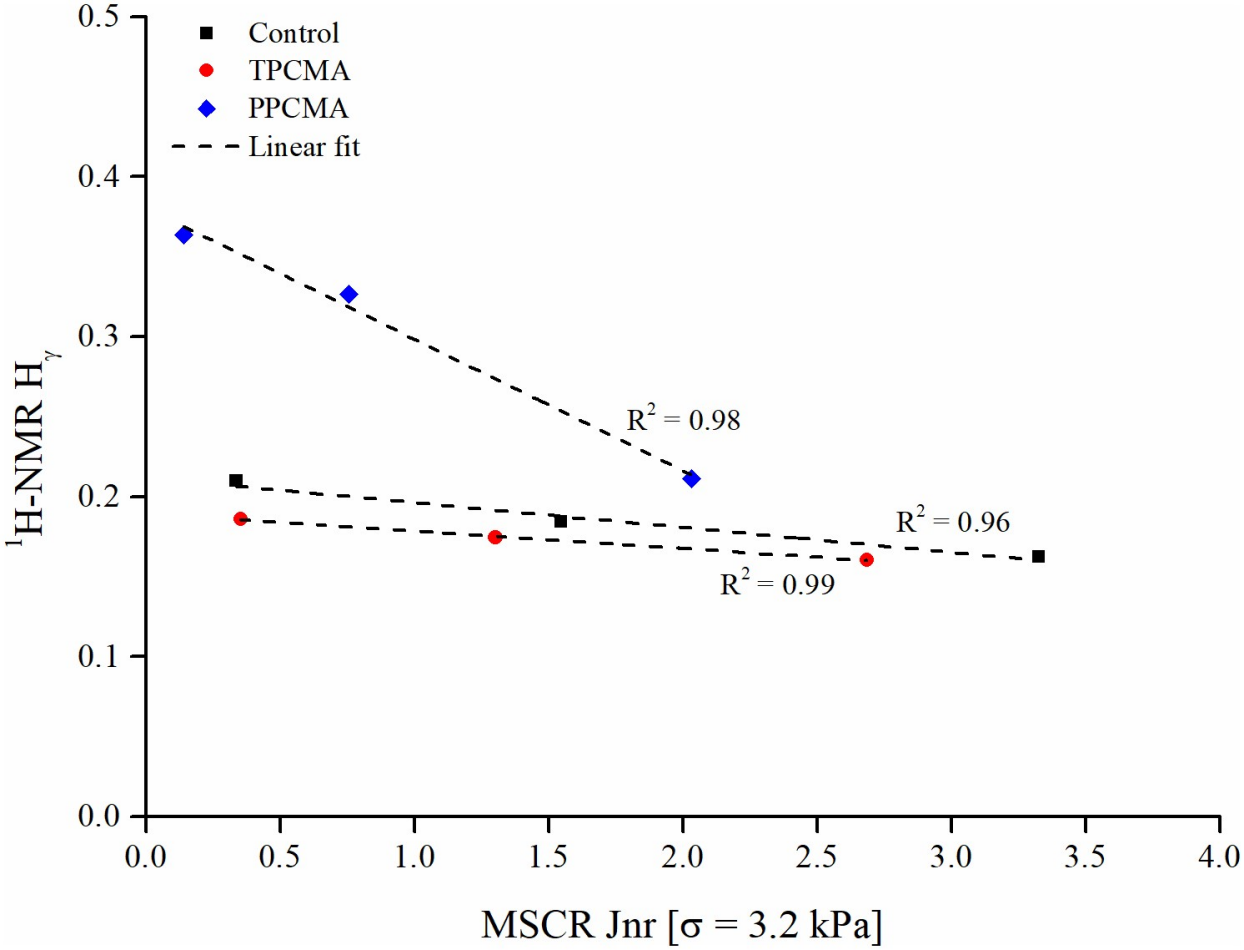
Correlation between MSCR *J*_*nr*_ and H_γ_.

The ranges of H_α_, H_β_, and H_γ_ were found as 0.010–0.153, 0.478–0.773, and 0.160–0.363, respectively. Among the three aliphatic protons (H_α_, H_β_, and H_γ_), the H_β_ represents the major saturated proton for all binders under all three aging states. This observation is also in agreement with those reported by [47,49,50]. No apparent changes could be determined for H_α_ and H_β_ with an increase in the degree of aging. However, a strong trend was found between H_γ_ and the severity of aging, with a greater H_γ_ value indicating to a more aged binder (Fig 17). Such a trend was also reported in [47], where higher H_γ_ values were found when the binder aging exposure period was increased from 30 days to 60, 90, and 120 days. Zhang and Hu [49] also observed a higher H_γ_ for the control (unmodified) and a crumb rubber-SBS-sulfur composite binder after the binders were subjected to thin film oven aging (another protocol that simulates short-term aging). H_γ_ describes aliphatic hydrogens in methyl (CH_3_) or methylene (CH_2_) groups in the γ position to an aromatic ring. In this study, H_γ_ was also found to have a strong negative correlation with the binder *J*_*nr*_ (3.2 kPa), as shown separately for the three binders in Fig 18. The correlation suggests that higher H_γ_ may be linked to the increase in binder rutting performance (indicated by a lower *J*_*nr*_). However, such a correlation may be specific to the control, TPC and PPC modified binders considered in this study, and further analysis with binders from other sources/modifiers is needed to generalize the finding.

## Conclusions

In this study, rheological and spectroscopic characterisation were used to assess the effect of aging on the properties of TPC and PPC modified asphalt binders. Four rheological aging indices were based on complex modulus (G*), phase angle (δ), zero shear viscosity (ZSV), and multiple stress creep and recovery (MSCR) tests conducted on a DSR. The fatigue cracking potential was also measured through binder yield energy test (BYET). Fourier transform infrared (FTIR) and proton nuclear magnetic resonance (^1^H-NMR) spectroscopic analyses were then used to investigate changes in chemical composition due to aging. Based on the results and analyses, the main conclusions drawn from the study are:

- Both TPC and PPC improved the high-temperature deformation resistance properties of asphalt binder as seen from the results of G*, δ, ZSV, and MSCR tests.
- Rheological indices based on the ratio of G*, δ, ZSV and *J*_*nr*_ showed that TPC-modified asphalt binder suffered the slightest aging compared to control and PPC-modified binders.
- FTIR results indicated that the addition of TPC retarded the carbonyl and sulfoxide chemical groups formed due to aging.
- Despite the observation that PPC-modified asphalt binder suffered more aging than other binders, it showed the highest binder yield energy at both test temperatures (15 and 25°C), indicating that the along with the increase in stiffness, it provided higher fatigue cracking resistance.
- ^1^H-NMR results indicated that aromatic hydrogen decreased with aging and aliphatic γ-hydrogen had strong correlations with binder *J*_*nr*_ in different aging states.

The research findings from this study provide a detailed insight on the changes due to aging on rheological and spectroscopic properties of TPC and PPC modified binders and their comparison to the control (unmodified) binder. The results obtained are quite encouraging in the direction of waste tire and plastic management using pyrolytic chars as sustainable asphalt binder modifiers. The conclusions drawn in the present study are based on the rheological and spectroscopic characteristics evaluated for the short-term and long-term aged asphalt binders modified with TPC and PPC, and further investigation at the level evaluation of asphalt mixtures is recommended for future works.

## Supporting information

S1 File Data. This file includes all the test data of the asphalt binders used in this study.
(XLSX)

## Abbreviations

G*: Complex shear modulus
*J*_*nr*_: Nonrecoverable creep compliance
*k* and *m*: Cross model constants
*δ*: Phase angle
*ε*_*nr*_: Non-recovered strain
*η**: Complex viscosity
*η*_*0*_: Zero shear viscosity
*η*_∞_: Viscosity at infinite frequency
*σ*: Stress
*ω*: Angular frequency

## References

[1] Martínez JD, Puy N, Murillo R, García T, Navarro MV, Mastral AM. Waste tire pyrolysis–a review. Renew. Sust. Energ. Rev. 2013; 23:179–213. doi: 10.1016/j.rser.2013.02.038

[2] Kunwar B, Cheng HN, Chandrashekaran SR, Sharma BK. Plastics to fuel: a review. Renew. Sust. Energ. Rev. 2016; 54: 421–428. doi: 10.1016/j.rser.2015.10.015

[3] Pinto F, Costa P, Gulyurtlu I, Cabrita I. Pyrolysis of plastic wastes: effect of plastic waste composition on product yield. J. Anal. Appl. Pyrolysis 1999; 51(1–2):39–55. doi: 10.1016/S0165-2370(99)00007-8

[4] Williams PT, Slaney E. Analysis of products from the pyrolysis and liquefaction of single plastics and waste plastic mixtures. Resour. Conserv. Recycl. 2007; 51(4): 754–769. doi: 10.1016/j.resconrec.2006.12.002

[5] Traxler RN. Relation between asphalt composition and hardening by volatilization and oxidation. Proc. Assoc. Asphalt Paving Technol. 1961; 30: 359–377.

[6] Yu X, Leng Z, Wei T. Investigation of the rheological modification mechanism of warm-mix additives on crumb-rubber-modified asphalt. J. Mater. Civ. Eng. 2014; 26(2): 312–319. doi: 10.1061/(ASCE)MT.1943-5533.0000808

[7] Petersen JC. Chemical composition of asphalt as related to asphalt durability: state of the art. Transp. Res. Rec. 1984; 999: 13–30.

[8] Sirin O, Paul DK., Kassem E. State of the art study on aging of asphalt mixtures and use of antioxidant additives. Adv. Civ. Eng. 2018. doi: 10.1155/2018/3428961

[9] Rahmani E, Darabi MK, Little DN, Masad EA. Constitutive modeling of coupled aging-viscoelastic response of asphalt concrete. Constr. Build. Mater. 2017; 131: 1–15. doi: 10.1016/j.conbuildmat.2016.11.014

[10] Huang Y, Bird RN, Heidrich O. A review of the use of recycled solid waste materials in asphalt pavements. Resour. Conserv. Recycl. 2007; 52(1): 58–73. doi: 10.1016/j.resconrec.2007.02.002

[11] Milad AA, Ali AS, Yusoff NI. A review of the utilisation of recycled waste material as an alternative modifier in asphalt mixtures. Civ. Eng. J. 2020; 6: 42–60. doi: 10.28991/cej-2020-SP(EMCE)-05

[12] Kumar A, Choudhary R, Narzari R, Kataki R, Shukla SK. Evaluation of bio-asphalt binders modified with biochar: a pyrolysis by-product of *Mesua ferrea* seed cover waste. Cogent Engineering. 2018; 5(1): 1548534. doi: 10.1080/23311916.2018.1548534

[13] Mawat HQ, Ismael MQ. Assessment of moisture susceptibility for asphalt mixtures modified by carbon fibers. Civil Engineering Journal. 2020; 6(2): 304–17. doi: 10.28991/cej-2020-03091472

[14] Aliotti AG. Carbon black-its nature and possible effects on the characteristics of bituminous road binders. In Australian Road Research Board (ARRB) Conference, 1st, 1962, Canberra 1962 (Vol. 1, No. 2).

[15] Lesueur D, Dekker L, Planche JP. Comparison of carbon black from pyrolized tires to other fillers as asphalt rheology modifiers. Transp. Res. Rec. 1995; 1515: 47–55.

[16] Feng ZG, Rao WY, Chen C, Tian B, Li X, Li PL, et al. Performance evaluation of bitumen modified with pyrolysis carbon black made from waste tires. Constr. Build. Mater. 2016; 111: 495–501. doi: 10.1016/j.conbuildmat.2016.02.143

[17] Li C, Fan Z, Wu S, Li Y, Gan Y, Zhang A. Effect of carbon black nanoparticles from the pyrolysis of discarded tires on the performance of asphalt and its mixtures. Appl. Sci. 2018; 8(4): 624. doi: 10.3390/app8040624

[18] Wang H, Lu G, Feng S, Wen X, Yang J. Characterization of bitumen modified with pyrolytic carbon black from scrap tires. Sustainability 2019; 11(6): 1631. doi: 10.3390/su11061631

[19] Kumar A, Choudhary R. Use of waste tire pyrolytic products for asphalt binder modification. Int. J. Pav. Eng. Asphalt Tech. 2020; 21: 35–51. doi: 10.1515ijpeat-2016-0031

[20] Kumar A, Choudhary R, Kumar A. Use of char derived from waste plastic pyrolysis for asphalt binder modification. In Int. Conf. Innov. Techn. Clean Sustain. Develop., RILEM Bookseries 2020; 29: 337–356. doi: 10.1007/978-3-030-51485-3_23

[21] Kumar A, Choudhary R, Kumar A. Use of waste plastic and tire pyrolytic char in asphalt binders: a sustainable approach for future pavements. Civil Eng. Const. Rev. 2020; 33(4): 22–26.

[22] IS 73. Paving Bitumen–Specification. Bureau of Indian Standards, New Delhi, 2013.

[23] ASTM D2872. Standard Test Method for Effect of Heat and Air on a Moving Film of Asphalt (Rolling Thin-Film Oven Test). ASTM International, West Conshohocken, PA, 2019.

[24] ASTM D6521. Standard Practice for Accelerated Aging of Asphalt Binder Using a Pressurized Aging Vessel (PAV). ASTM International, West Conshohocken, PA, 2019.

[25] ASTM D7405. Standard Test method for multiple stress creep and recovery (MSCR) of asphalt binder using a dynamic shear rheometer. ASTM International, West Conshohocken, PA, 2020.

[26] Johnson C, Bahia H, Wen H. Practical application of viscoelastic continuum damage theory to asphalt binder fatigue characterization. J. Assoc. Asphalt Paving Technol. 2009; 28: 597–638.

[27] Kumar A, Choudhary R, Kandhal PS, Julaganti A, Behera OP, Singh A, Kumar R. Fatigue characterisation of modified asphalt binders containing warm mix asphalt additives. Road Mater. Pavement Des. 2020; 21(2): 519–541. doi: 10.1080/14680629.2018.1507921

[28] AASHTO TP 123. Standard method of test for measuring asphalt binder yield energy and elastic recovery using the dynamic shear rheometer. Washington, DC: American Association of State Highway and Transportation Officials, 2016.

[29] Wang F, Xiao Y, Cui P, Lin J, Li M, Chen Z. Correlation of asphalt performance indicators and aging degrees: A review. Constr. Build. Mater. 2020; 250: 118824. doi: 10.1016/j.conbuildmat.2020.118824

[30] Iqbal M, Hussain A, Khattak A, Ahmad K. Improving the aging resistance of asphalt by addition of polyethylene and sulphur. Civ. Eng. J. 2020; 6(5): 1017–30. doi: 10.28991/cej-2020-03091525

[31] Brown ER, Kandhal PS, Roberts FL, Kim YR, Lee DY, Kennedy TW. Hot mix asphalt materials, mixture design, and construction. NAPA Research and Education Foundation; 2009.

[32] Jin D, Ge D, Chen S, Che T, Liu H, Malburg L, et al. Cold in-place recycling asphalt mixtures: laboratory performance and preliminary ME design analysis. Materials. 2021; 14(8): 2036. doi: 10.3390/ma14082036 33919543 PMC8074015

[33] Williams PT. Pyrolysis of waste tires: A review. Waste Manag. 2013; 33(8): 1714–1728. doi: 10.1016/j.wasman.2013.05.003 23735607

[34] Helleur R, Popovic N, Ikura M, Stanciulescu M, Liu D. Characterization and potential applications of pyrolytic char from ablative pyrolysis of used tires. J. Anal. Appl. Pyrolysis 2001; 58: 813–824. doi: 10.1016/S0165-2370(00)00207-2

[35] Apeagyei K. Laboratory evaluation of antioxidants for asphalt binders. Constr. Build. Mater. 2011; 25(1): 47–53. doi: 10.1016/j.conbuildmat.2010.06.058

[36] Chaala A, Roy C, Ait-Kadi A. Rheological properties of bitumen modified with pyrolytic carbon black. Fuel. 1996; 75(13): 1575–83. doi: 10.1016/0016-2361(96)00143-3

[37] Jamradloedluk J, Lertsatitthanakorn C. Characterization and utilization of char derived from fast pyrolysis of plastic wastes. Procedia Eng. 2014; 69: 1437–1442. doi: 10.1016/j.proeng.2014.03.139

[38] Saptoadi H, Rohmat TA, Sutoyo. Combustion of char from plastic wastes pyrolysis. In AIP Conf. Proc. 2016; 1737.

[39] Lu X, Isacsson U. Effect of ageing on bitumen chemistry and rheology. Constr. Build. Mater. 2002; 16: 15–22. doi: 10.1016/S0950-0618(01)00033-2

[40] Lamontagne J, Dumas P, Mouillet V, Kister J. Comparison by Fourier transform infrared (FTIR) spectroscopy of different ageing techniques: application to road bitumens. Fuel 2001; 80(4): 483–488. doi: 10.1016/S0016-2361(00)00121-6

[41] Singh B, Saboo N, Kumar P. Use of Fourier transform infrared spectroscopy to study ageing characteristics of asphalt binders. Pet. Sci. Technol. 2007; 35(16): 1648–1654. doi: 10.1080/10916466.2017.1350710

[42] Qian G, Yu H, Jin D, Bai X, Gong X. Different water environment coupled with ultraviolet radiation on ageing of asphalt binder. Road Mater. Pavement Des. 2020; 1–14. doi: 10.1080/14680629.2020.1760920

[43] Nivitha MR, Prasad E, Krishnan JM. Ageing in modified bitumen using FTIR spectroscopy. Int. J. Pavement Eng. 2016; 17(7): 565–577. doi: 10.1080/10298436.2015.1007230

[44] Nciri N, Kim N, Cho N. New insights into the effects of stirene-butadiene-stirene polymer modifier on the structure, properties, and performance of asphalt binder: The case of AP-5 asphalt and solvent deasphalting pitch. Mater. Chem. Phys. 2017; 193: 477–495. doi: 10.1016/j.matchemphys.2017.03.014

[45] Rossi CO, Caputo P, De Luca G, Maiuolo L, Eskandarsefat S, Sangiorgi C. ^1^H-NMR spectroscopy: a possible approach to advanced bitumen characterization for industrial and paving applications. Appl. Sci. 2018; 8(2): 229. doi: 10.3390/app8020229

[46] Redelius P, Soenen H. Relation between bitumen chemistry and performance. Fuel 2015; 140: 34–43. doi: 10.1016/j.fuel.2014.09.044

[47] Ma L, Li Z, Huang J. Investigation of chemistry by FTIR and NMR during the natural exposure aging of asphalt. In Pavements and materials: Recent advances in design, testing and construction 2011; 150–157. doi: 10.1061/47623(402)18

[48] Xu M, Zhang Y, Zhao P, Liu C. Study on aging behavior and prediction of SBS modified asphalt with various contents based on PCA and PLS analysis. Constr. Build. Mater. 2020; 265: 120732. doi: 10.1016/j.conbuildmat.2020.120732

[49] Zhang F, Hu C. The research for structural characteristics and modification mechanism of crumb rubber compound modified asphalts. Constr. Build. Mater. 2015; 76: 330–342. doi: 10.1016/j.conbuildmat.2014.12.013

[50] Sun D, Yu F, Li L, Lin T, Zhu XY. Effect of chemical composition and structure of asphalt binders on self-healing. Constr. Build. Mater. 2017; 133: 495–501. doi: 10.1016/j.conbuildmat.2016.12.082

